# Exercise and Sports Among Working-Age Citizens in Lithuania Since the COVID-19 Pandemic: An Annual Comparative Study (2021–2024)

**DOI:** 10.3390/medicina62010131

**Published:** 2026-01-08

**Authors:** Rokas Arlauskas, Donatas Austys, Rimantas Stukas, Valerij Dobrovolskij, Arūnas Rimkevičius, Gabija Bulotaitė

**Affiliations:** 1 Department of Public Health, Institute of Health Sciences, Faculty of Medicine, Vilnius University, M. K. Čiurlionio 21/27, LT-03101 Vilnius, Lithuania; donatas.austys@mf.vu.lt (D.A.); rimantas.stukas@mf.vu.lt (R.S.); valerij.dobrovolskij@mf.vu.lt (V.D.); gabija.bulotaite@mf.vu.lt (G.B.); 2 Institute of Odontology, Faculty of Medicine, Vilnius University, Žalgirio 115, 117, LT-08215 Vilnius, Lithuania; arunas.rimkevicius@mf.vu.lt

**Keywords:** physical activity, exercise, sports, adults, working-age, Lithuania, prevalence

## Abstract

*Background and Objectives:* The COVID-19 pandemic had a significant impact on physical activity among various populations. Due to a lack of country-representative studies on the prevailing trends in leisure-time physical activity since the COVID-19 pandemic, the aim of this study was to assess the temporal, social, and demographic inequalities in the prevalence of engagement in exercise and sports among working-age citizens of Lithuania from 2021 to 2024. *Materials and Methods:* This study included four samples of working-age citizens (1600 per year, 6400 in total). Four surveys were conducted, and the distribution of respondents among the groups was compared. *Results:* In general, the prevalence of engagement in exercise and sports did not change over a four-year period (48.8%, *p* = 0.256). The prevalence of regular exercise and sports increased, while engagement in irregular exercise and sports decreased (*p* = 0.014). Binary logistic regression analysis showed that younger age, male sex, being single, having no children under 18 years of age, selecting foods for health strengthening, positive self-assessment of nutrition and health status, use of dietary supplements, attention to purchasing healthy products, and university education attainment were associated with engagement in exercise and sports (regular or irregular) (*p* < 0.05). Analysis focused specifically on regular exercise and sports revealed associations with a longer time since the onset of the COVID-19 pandemic, younger age, urban residence, selection of foods for health strengthening, positive assessment of nutrition and health status, and university education attainment (*p* < 0.05), while no significant associations were observed with sex, marital status, presence of children under 18 years of age, use of dietary supplements, or attention to purchasing healthy products (*p* > 0.05). *Conclusions:* The overall prevalence of physical activity engagement among working-aged Lithuanian citizens did not change from 2021 to 2024, engagement in regular and irregular exercise and sports has changed. Engagement in regular and irregular exercise and sports is associated with different social profiles.

## 1. Introduction

Multiple studies have shown that the COVID-19 pandemic had a significant impact on the lifestyle of various populations [1,2,3,4,5,6,7,8,9]. Even though some of them revealed the occurrence of positive changes [1,6,7,8], there were studies highlighting unfavorable lifestyle changes [1,2,3,4,5,9].

The COVID-19 pandemic may have contributed to the long-lasting prevalence of insufficient physical activity registered in many populations worldwide [10]. Studies show that despite the recovery of physical activity after the pandemic restrictions, some residual effects remained, and lower levels of physical activity were observed [3,4,5]. Apparently, several social changes triggered by the pandemic have become integral to our daily lives. Those changes present both risks and opportunities for physical activity [11] and should be taken into account when planning the interventions for physical activity promotion. Furthermore, it was shown that the pandemic-induced changes in the prevalence of physical activity differ not only among various social and demographic groups (e.g., sex, age, socioeconomic status, marital status, education) [3,4] but also among the groups with different intensities of physical activity [2,5].

In Lithuania, as in many other countries, one of the recommended protective measures against the spread of the COVID-19 disease was the restriction of physical contact due to the fact that the virus spreads from person to person via airborne transmission [12]. During the COVID-19 pandemic, the duration and severity of restrictions varied between countries [13]. Moreover, public attitude and compliance with restrictions were different [14]. Additionally, there were recommendations to stay as active as possible during the COVID-19 pandemic [15,16,17,18,19]; the COVID-19 period contributed to inactivity by forcing people to stay at home and restricting their freedom to participate in sports [20,21,22]. In Lithuania, quarantine restrictions were implemented several times in response to COVID-19 incidence rates. The first national quarantine began on 16 March 2020 and lasted approximately three months. After a four-month interval, a second quarantine was introduced on 7 November 2020. Following a gradual easing of restrictions, it was lifted in mid-2021. Since then, no further national quarantines have been imposed, aside from requirements for personal protective measures and testing. Despite the absence of strict restrictions for professional athletes, the vast majority of the Lithuanian population faced limitations on exercising and sports participation, as group activities were not permitted [23,24].

Despite the fact that previous studies have examined various factors as the determinants of physical activity of people from various demographic groups, there is a lack of country-representative studies on the ongoing trends in the prevalence of leisure-time physical activity of various intensities since the COVID-19 pandemic. Taking this into account, the aim of this study was to assess the temporal, social, and demographic inequalities in the prevalence of engagement in exercise and sports among the working-age citizens of Lithuania from 2021 to 2024.

## 2. Materials and Methods

### 2.1. Data Collection

The data for this study were collected after conducting four independent cross-sectional surveys in the months of October and November of 2021, 2022, 2023, and 2024. A representative sample of adults aged 18 to 64 was formed each year. The multistage stratified probabilistic sampling method was used to select the participants for this study. It ensured an equal probability for every household in the country to be surveyed, and, according to target criteria (sex, age, municipality, education, income, employment, marital status), the sample represented the general population of working-age citizens of Lithuania. Data were collected by conducting an internet-based survey. Random samples of citizens were formed according to the Registry of Residents of Lithuania. Every selected citizen received an invitation to participate in this study with a link to the anonymous questionnaire by email. The study participants filled out the questionnaire by themselves at a time that was convenient to them. The questionnaire could be completed only a single time per year. Only the working-age citizens of Lithuania were included in this study. This study did not include refugees or individuals without Lithuanian citizenship.

Each of the samples independently included 1600 citizens. The design of this study was not longitudinal. No data about the inclusion of the respondents in more than one sample was collected. In total, this paper deals with the responses obtained from 6400 respondents.

This study was reviewed by the Vilnius Regional Biomedical Research Ethics Committee.

### 2.2. Description of the Questionnaire

Each of the four surveys was carried out using the same questionnaire with a minimal adaptation for the post-pandemic period. An anonymous questionnaire included questions about the social and demographic characteristics of the respondents, the severity of the COVID-19 cases among respondents, their subjective assessment of personal health, nutrition, consumption of food supplements, and physical activity. The questionnaire was formed on the basis of the previously used questionnaire about nutrition and the consumption of food supplements [8]. Apart from the questions concerning the respondents’ social and demographic characteristics (sex, age, education, the place of residence, marital status, the number of family members, the number of children in the household, employment status, and income level), this article examines the respondents’ answers to the questions related to respondents’ health behavior and lifestyle presented in Table 1.

Two of the questions regarding the respondents’ age and place of residence were open-ended. To achieve the unambiguous interpretation of the results, we transformed them into a binary format. Respondents were asked to identify the municipality they lived in. Respondents from 5 municipalities with the largest number of residents were assigned to the “City” group, while the remaining respondents were assigned to the “Towns and Villages” group. The age was categorized by median, up to 41 years old, and from 42 years old individuals. All other questions were closed. Respondents with primary or secondary education and high school graduates were assigned to the “non-university education” group. Respondents with unfinished or finished university studies were assigned to the “University education” group. In terms of employment status, the “Employed” and “Unemployed” groups were created. Heads of companies or departments, office workers, civil servants, service sector employees, sellers, workers, and farmers were assigned to the “Employed” group. Retirees, housewives, individuals on parental leave, non-employed individuals, and students were categorized into the “Unemployed” group. The variable representing an income per member of a family was transformed into a binary format with “higher income” and “lower income” categories. With respect to economic changes in Lithuania, the cut-off point for those groups was 350 EUR in 2021, 400 EUR in 2022, 470 EUR in 2023, and 480 EUR in 2024. In addition to this, more binary variables were created, such as the number of family members, marital status, and families with children under 18 years old. The categorization of the rest of the questionnaire is presented in Table 2.

### 2.3. Statistical Analysis

The normality of the variable representing the age of respondents in general samples was tested using the Kolmogorov–Smirnov test with the Lilliefors significance correction. This test showed non-normal distributions. Therefore, medians with an interquartile range (Q1–Q3) were presented for this variable. Pearson’s chi-squared test (χ^2^) was used to determine whether there were statistically significant differences between the expected frequencies and the observed frequencies in one or more of the categories. Differences were considered statistically significant when the *p*-value was lower than 0.05. Additionally, two binary logistic regression models were constructed: one to predict general engagement in exercise and sports (including regular and irregular participation) and another to predict regular engagement. Only variables with *p*-values up to 0.1 were retained in the final models. Odds ratios and 95% confidence intervals were calculated for each variable. The Hosmer–Lemeshow goodness-of-fit test was applied. For the logistic regression models, variables were selected using forward and backward conditional procedures. The variable on COVID-19 severity was excluded due to a high proportion of missing values. Age and number of family members were included as continuous variables.

## 3. Results

The distribution of the respondents among the samples of 2022, 2023, and 2024 was similar in terms of sex, age, the education level, the type of place of residence, the number of family members, the presence of children in the household, employment status, the severity of COVID-19, the subjective assessment of health status, overall engagement in exercise and sports (*p* > 0.05). The first two samples consisted of a significantly larger proportion of single respondents (*p* < 0.05). In the last sample of 2024, an increase in the prevalence of lower income per family member was observed (*p* < 0.05). In 2022, a significant increase in the prevalence of food selection for other reasons than to strengthen health was found, while in 2023 and 2024, there was a significant increase in the prevalence of food selection to strengthen health (*p* < 0.05). In 2022, there was a rise in both negative subjective assessment of nutrition and in the consumption of dietary supplements, while in the upcoming years these groups remained unchanged (*p* < 0.05). A fluctuating pattern with a decline in 2022 and a recovery in 2023 was found in those who pay attention to buying healthy products (*p* < 0.05). In 2024, those engaged in regular exercise and sports took a bigger part of the sample (*p* < 0.05) (Table 2).

**Figure 1:**
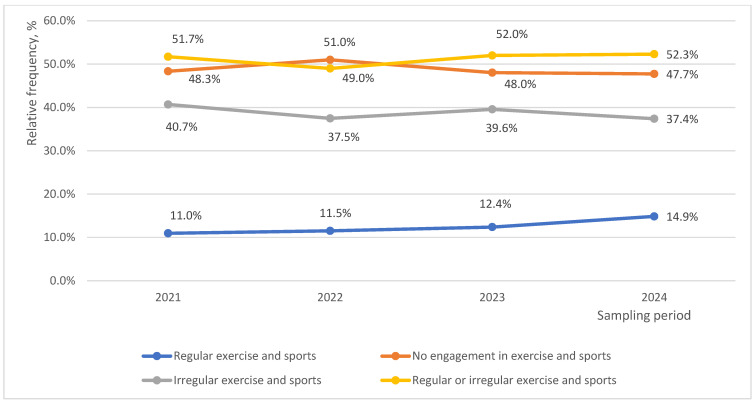
Prevalence of engagement in regular and irregular exercise and sports.

In general, the engagement in exercise and sports remained unchanged over the period of four years (48.8%, *p* = 0.256). The comparison of the proportions of the respondents engaged in regular and irregular exercise and sports over the period of four years revealed an increase in regular exercise and sports and a decrease in irregular exercise and sports (*p* = 0.014) (Figure 1).

Among those who were not engaged in either regular or irregular exercise and sports, there were no differences in the distribution between the samples in terms of sex, age, education level, the type of place of residence, the number of family members, the presence of children in the household, employment status, income level, the severity of COVID-19, the subjective assessment of health status, the subjective assessment of nutrition (*p* > 0.05). The first two samples consisted of a significantly larger proportion of single respondents (*p* < 0.05). In 2022, food selection for other reasons than strengthening health was significantly more prevalent, but food selection to strengthen health significantly prevailed, and the prevalence of negative assessment of health status increased in 2023 (*p* < 0.05). In 2022, the prevalence of the consumption of dietary supplements dropped (*p* < 0.05) and remained at that level until 2024. The proportion of those who pay attention to buying healthy products declined in 2022 and returned to the previous level in 2023 (*p* < 0.05), remaining unchanged until 2024 (Table 3).

The distribution patterns of the respondents engaged in regular or irregular exercise and sports in all four samples did not differ in terms of sex, age, the type of place of residence, the number of family members, the presence of children in the household, income level, the severity of COVID-19, the subjective assessment of health status, health-consciousness in product choice (*p* > 0.05). The proportion of those with university education decreased in 2022 and remained unchanged until 2024 (*p* < 0.05). The first two samples comprised a significantly larger number of single respondents (*p* < 0.05). The proportion of employed respondents decreased in 2022 and returned to the previous level in 2023 (*p* < 0.05). In 2022, a significant rise in the prevalence of food selection for other reasons than health strengthening was observed, while in 2023, physically active respondents significantly more often opted for food selection to strengthen health (*p* < 0.05). Over the period of four years, there was a significant decline in the number of those who positively assessed their nutrition (*p* < 0.05). The consumption of dietary supplements dropped in 2022 before returning to the previous level in 2023 (*p* < 0.05) (Table 4).

The comparison of the samples showed no differences in the distribution of respondents engaged in regular exercise and sports in terms of sex, age, the type of place of residence, the number of family members, the presence of children in the household, the severity of COVID-19, the subjective assessment of health status, the subjective assessment of nutrition, health-consciousness in product choice (*p* > 0.05). The proportion of those with university education decreased in 2022 and remained unchanged until 2024 (*p* < 0.05). The first two samples included a significantly higher share of single respondents (*p* < 0.05). The proportion of employed respondents and those with higher income decreased in 2022 but returned to the previous levels in 2023 (*p* < 0.05). In 2022, a significant rise in the prevalence of food selection for other reasons than health strengthening was observed (*p* < 0.05). Despite the fluctuation in 2023 (*p* < 0.05), the overall prevalence of the consumption of dietary supplements was similar among the four samples (*p* > 0.05) (Table 5).

In at least three of the samples, respondents with university education, higher income, the positive assessment of health status and nutrition, employed respondents, those who selected foods to strengthen health or due to dietary necessity, consumed dietary supplements, paid attention to buying healthy products, and more frequently engaged in regular or irregular exercise and sports (*p* < 0.05). In at least two of the samples, a more frequent engagement in regular or irregular exercise and sports was observed among males, those without children, urban, and single respondents (*p* < 0.05). In all samples, there was no association between the engagement in exercise and sports (regular or irregular) and age, the number of family members, and the severity of COVID-19 (*p* > 0.05) (Table 6).

Binary logistic regression analysis revealed that being younger, male, single, without children under 18 years of age, selecting foods for health strengthening, having a positive assessment of nutrition, a positive self-assessment of health status, using dietary supplements, paying attention to purchasing healthy products, and having a university education were associated with engagement in exercise and sports (regular or irregular) (Table 7).

In at least three of the samples, respondents with university education, higher income, the positive assessment of health status and nutrition, younger, urban respondents, those who selected foods to strengthen health or for dietary necessity, and those who paid attention to buying healthy products more frequently engaged in regular exercise and sports (*p* < 0.05). Additionally, in 2023, the higher prevalence of regular exercise and sports was observed among those who consumed dietary supplements (*p* < 0.05). Overall, the four consecutive years, no association was detected between the respondents’ regular exercise and sports and sex, marital status, the number of family members, the presence of children in the household, employment, and the severity of COVID-19 (*p* > 0.05) (Table 8).

Binary logistic regression analysis revealed that a longer time since the onset of the COVID-19 pandemic, younger age, urban residence, selecting foods for health strengthening, a positive assessment of nutrition, a positive self-assessment of health status, and university education attainment were associated with engagement in regular exercise and sports (Table 9).

## 4. Discussion

The results of this study have revealed that the overall engagement in exercise and sports in the working-age population of Lithuania did not change over a four-year period, while the prevalence of regular exercise and sports increased, and the prevalence of irregular exercise and sports decreased. The main facilitating factors for exercise and sports are likely to be university education, younger age, and the selection of foods to strengthen health or dietary necessity. Distinct differences were found between the engagement in the general exercise and sports and regular exercise and sports profiles with respect to sex, marital status, place of residence, the presence of children in the household, use of dietary supplements, and health-consciousness in product choice. Male, single (without a partner or children) individuals, and those buying healthy products and using dietary supplements tend to engage in general exercise and sports, while urban citizens of Lithuania are likely be more frequently engaged in regular exercise and sports. Even though a decrease in the prevalence of higher income and a positive assessment of nutrition was observed in the overall sample, including both physically active and inactive respondents, no notable changes with respect to these factors were observed among those not engaged in exercise and sports.

Similar trends were prevailing among three groups of respondents—those not engaged in exercise and sports, those engaged in regular or irregular exercise and sports, and those engaged in regular exercise and sports—with respect to sex, age, the type of place of residence, the number of family members, the presence of children in the household, the severity of COVID-19, the subjective assessment of health status, marital status, and food selection criteria. In all four samples, both groups of engaged in exercise and sports respondents showed to be consistent in terms of health-consciousness in product choice, while those with no engagement in exercise and sports showed fluctuations of this factor. Respondents engaged in regular exercise and sports, and those not engaged in exercise and sports consistently gave positive subjective assessments of nutrition, as seen in four samples. However, the number of those engaged in irregular exercise and sports declined. The overall prevalence of the consumption of dietary supplements was similar among those engaged in regular exercise and sports across all four samples, and declining or fluctuating patterns were observed among those with irregular or not engaged in exercise and sports. With respect to employment status and income level, no changes across the samples were found among those with no engagement in exercise and sports. However, among those with regular exercise and sports, the proportion of employed respondents and those with higher income decreased in 2022 and returned to the previous levels in 2023. According to the education level, there were no changes among those not engaged in exercise and sports. However, the proportion of those with a university degree among those with regular or irregular physical activity decreased in 2022 and remained unchanged until 2024.

Our findings that only minor changes in engagement in exercise and sports occurred because of the COVID-19 pandemic are similar to the results of another cross-sectional study conducted in Lithuania in 2024. Despite the differences in the age groups, it was found that the prevalence of physical activity among the majority of older adults in Lithuania did not change because of the pandemic or returned to the pre-pandemic level [25]. On the other hand, our results were slightly contradictory to those obtained from a longitudinal study regarding the long-term effects of the COVID-19 pandemic on the physical activity of the Italian population. It was revealed that there were no significant differences among the physical activity levels between the pre- and post-pandemic periods in the Italian population [5], while there was a rise in the prevalence of regular exercise and sports in the Lithuanian population after the pandemic. It may not necessarily mean that the intensity of physical activity of the Lithuanian citizens increased, but regular exercise and sports often require a higher intensity of physical activity.

Similarly to the Italian study carried out by Bifolco et al., the observations of physical activity of South American adults [26] and pregnant women in the USA [2] showed a significant decrease during the COVID-19 pandemic. Our results revealed no significant differences when comparing the prevalence of overall exercise and sports during and after the pandemic.

Similarly to the study that was conducted in Spain, our results show that people who are engaged in exercise and sports more frequently assess their health positively. In the Spanish population, the correlation between the time spent performing sports was positively correlated with the self-perceived health during and after the COVID-19 pandemic [27]. This might also spotlight another of our findings, stating that people with regular exercise and sports show a rarely changing profile, which is hardly affected by various factors.

Considering exercise and sports as a social behavior, as suggested by Wang et al. [28], the results of our study might be beneficial for the development of targeted interventions for the promotion of engagement in exercise and sports. Our results spotlighted a list of social and demographic factors indicating groups of working-aged Lithuanians with engagement in regular exercise and sports, engagement in overall (regular or irregular) exercise and sports, and no engagement in exercise and sports. These lists of social and demographic factors might be beneficial for understanding the reasons for non-engagement in regular exercise and sports, and what is likely to motivate our modern society to engage in exercise and sports.

### Limitations

The country-wide cross-sectional design of this study allowed us to assess only the subjective views of the respondents on engagement in regular and irregular exercise and sports. A longitudinal study would have enabled us to objectively assess the changes in the frequency and the intensity of physical activities at an individual level. In addition, this would have allowed us to use more advanced statistical methods, facilitating the prediction of changes in the prevalence and intensity of exercise and sports.

In addition, the annual samples were large, and multistage probability sampling was used; data were collected via online surveys, which could introduce selection bias, as only individuals with email access, internet connectivity, and willingness to respond were included.

Moreover, in order to simplify the presentation and the interpretation of the results, we converted the age-representing variable into a binary variable. Despite the fact that it revealed some significant differences in the prevalence of engagement in exercise and sports, such conversion may hide some subgroups that may significantly differ from each other. To address this limitation, we additionally performed a logistic regression analysis treating age as a continuous variable, which allowed us to account for age-related variation more precisely. However, in the upcoming studies, it would be beneficial to perform a more detailed analysis.

Despite the fact that we analyzed the prevalence of engagement in physical activity with respect to many social, demographic, and health-related factors, there might be important factors that were not included in our analysis.

Due to limitations on the length of our questionnaire, we were unable to include additional questions on the frequency, duration, intensity, or work/leisure domains of physical activity. Consequently, physical activity was assessed using a single self-reported question with three broad categories, which limits comparability with international guidelines and may be affected by reporting bias.

## 5. Conclusions

Lithuanian working-age citizens have different social profiles in terms of their engagement in physical activity; while male, single, and without children individuals tend to engage in general exercise and sports, engagement in regular exercise and sports is associated with urban residence. The main facilitating factors for exercise and sports are university education, younger age, and the selection of foods to strengthen health or dietary necessity. Since the COVID-19 pandemic, the group with regular exercise and sports has shown less frequently changing associations with certain social and demographic factors.

## Figures and Tables

**Table 1:** Questions and response options related to respondents’ health behavior and lifestyle.

Question	Categories with Relevant Response Options *
Do you consume dietary supplements (vitamins, minerals, polyunsaturated fatty acids, plant-based preparations, etc.)?	Yes (yes, always/yes, more than 6 months per year/yes, 4–6 months per year/yes, 2–3 months per year/yes, 1 month per year/yes, but shortly or accidentally) No (no, I do not consume) Excluded from the analysis (I do not know/cannot answer)
What is the most important for you when selecting food products?	Health strengthening (Benefits to health) Other (Taste/Price/Preferences of other family members/The necessity of diet/Other) Excluded from the analysis (I do not know/cannot answer)
How would you assess your nutrition?	Positively (Very good/Rather good/Neither good nor bad) Negatively (Rather bad/Very bad) Excluded from the analysis (I do not know/cannot answer)
Is it important to you that food products are healthy, natural, organic, and free from artificial additives?	Pay attention to buying healthy products (I always pay attention to the food products I buy and look only for healthy options/I sometimes pay attention to the descriptions or labels about healthiness or naturalness of the food products I purchase) Do not care about the healthiness of products (I do not bother about what is written on food packaging; I simply buy what I need)
How would you assess your health?	Positively (Very good/Rather good/Neither good nor bad) Negatively (Rather bad/Very bad) Excluded from the analysis (I do not know/cannot answer)
Please select the most appropriate statement about your COVID-19 infection:	Suffered from an asymptomatic or a mild form of COVID-19 (I had an asymptomatic form of this disease/I had a mild form of this disease)/Suffered from a severe COVID-19 form (I had a severe form of this disease/I had a very severe form of this disease)
How would you assess your physical activity?	Engagement in regular exercise and sports (I engage in regular exercise or sports and attend training sessions systematically) Engagement in irregular exercise and sports (I engage in exercise or sports during my leisure time (irregularly, occasionally, or a few times per month)) No engagement in exercise and sports (I do not engage in exercise or sports activities) Excluded from the analysis (I do not know/cannot answer)

* In the case of broader categories, the response options are provided in brackets.

**Table 2:** Distribution of the respondents by social and demographic factors in four samples.

Factor	Sample of 2021		Sample of 2022		Sample of 2023		Sample of 2024		*p*-Value
	*n*	%	*n*	%	*n*	%	*n*	%	
**Sex**	1600		1600		1600		1600		0.957
Male	792	49.5%	800	50.0%	785	49.0%	788	49.3%	
Female	808	50.5%	800	50.0%	815	51.0%	812	50.8%	
**Age**	1600		1600		1600		1600		0.752
41 years old or younger	769	48.1%	784	49.0%	753	47.1%	768	48.0%	
42 years old or older	831	51.9%	816	51.0%	847	52.9%	832	52.0%	
**Education**	1484		1495		1506		1509		0.065
Non-university education	474	31.9%	495	33.1%	465	30.9%	532 ^	35.3% ^	
University education	1010	68.1%	1000	66.9%	1041	69.1%	977 *	64.7% *	
**Place of residence**	1600		1600		1600		1600		0.949
A small town or village	922	57.6%	914	57.1%	930	58.1%	918	57.4%	
City	678	42.4%	686	42.9%	670	41.9%	682	42.6%	
**Marital status**	1600		1600		1600		1600		<0.001 **
Married	964	60.2%	956	59.8%	1092 ^	68.2% ^	1074	67.1%	
Single	636	39.8%	644	40.2%	508 *	31.8% *	526	32.9%	
**Number of family members**	1600		1600		1600		1600		0.150
Two or more	1408	88.0%	1370 *	85.6% *	1369	85.6%	1378	86.1%	
One	192	12.0%	230 ^	14.4% ^	231	14.4%	222	13.9%	
**With children under 18 years old**	1600		1600		1600		1600		0.306
Yes	609	38.1%	633	39.6%	610	38.1%	654	40.9%	
No	991	61.9%	967	60.4%	990	61.9%	946	59.1%	
**Employment**	1494		1471		1480		1467		0.542
Employed	1174	78.6%	1135	77.1%	1139	76.9%	1155	78.7%	
Unemployed	321	21.4%	336	22.9%	341	23.1%	312	21.3%	
**Income**	1248		1226		1275		1278		0.026 **
Higher	836	67.0%	789	64.3%	866	67.9%	803 *	62.8% *	
Lower	412	33.0%	437	35.7%	409	32.1%	475^	37.2%^	
**Food selection criteria**	1569		1541		1576		1573		<0.001 **
Other	1081	68.9%	1256 ^	81.5% ^	1195 *	75.8% *	1129 *	71.8% *	
Health strengthening	489	31.1%	286 *	18.5% *	381 ^	24.2% ^	444 ^	28.2% ^	
**Severity of COVID-19**	343		385		385		349		0.053
Suffered from an asymptomatic or a mild form of COVID-19	264	77.1%	293	76.2%	287	74.4%	240	68.8%	
Suffered from a severe form of COVID-19	78	22.9%	92	23.8%	99	25.6%	109	31.2%	
**Subjective assessment of health status**	1587		1569		1570		1600		0.104
Positive	1472	92.7%	1433	91.3%	1419	90.4%	1471	91.9%	
Negative	115	7.3%	136	8.7%	151	9.6%	129	8.1%	
**Subjective assessment of nutrition**	1596		1577		1573		1571		0.002 **
Positive	1419	88.9%	1349 *	85.5% *	1339	85.1%	1332	84.8%	
Negative	177	11.1%	228 ^	14.5% ^	234	14.9%	239	15.2%	
**Consumption of dietary supplements**	1587		1579		1573		1592		<0.001 **
No	347	21.9%	448^	28.4%^	410	27.3%	432	27.1%	
Yes	1240	78.1%	1131 *	71.6%*	1163	72.7%	1160	72.9%	
**Health-consciousness in product choice**	1600		1600		1600		1600		<0.001 **
Pays attention to buying healthy products	1315	82.2%	1166 *	72.9% *	1266 ^	79.1% ^	1261	78.8%	
Does not care about the healthiness of products	285	17.8%	434 ^	27.1% ^	334 *	20.9% *	339	21.2%	
**Engages in exercise and sports (regular or irregular)**	1556		1516		1521		1517		0.146
No	746	47.9%	772	50.9%	721	47.4%	717	47.3%	
Yes	810	52.1%	744	49.1%	800	52.6%	800	52.7%	
**Engages in regular exercise and sports**	1556		1516		1521		1517		0.006 **
No	1381	88.8%	1332	87.9%	1330	87.4%	1285 *	84.7% *	
Yes	175	11.2%	184	12.1%	191	12.6%	232 ^	15.3% ^	

^ a significantly higher prevalence (*p* < 0.05) compared to the sample collected a year ago (2021 vs. 2022, 2022 vs. 2023, 2023 vs. 2024); * a significantly lower prevalence (*p* < 0.05) compared to the sample collected a year ago (2021 vs. 2022, 2022 vs. 2023, 2023 vs. 2024); ** a significant difference (*p* < 0.05) among all four samples (2021, 2022, 2023, and 2024).

**Table 3:** Distribution of the respondents not engaged in exercise and sports (regular or irregular) by social and demographic factors in four samples.

Factor	Sample of 2021		Sample of 2022		Sample of 2023		Sample of 2024		*p*-Value
	*n*	%	*n*	%	*n*	%	*n*	%	
**Sex**									0.627
Male	348	46.6%	370	47.9%	324	44.9%	324	45.2%	
Female	398	53.4%	402	52.1%	397	55.1%	393	54.8%	
**Age**									0.764
41 years old or younger	352	47.2%	383	49.6%	342	47.4%	349	48.7%	
42 years old or older	394	52.8%	389	50.4%	379	52.6%	368	51.3%	
**Education**									0.491
Non-university education	254	37.0%	261	36.6%	241	35.4%	266	39.3%	
University education	432	63.0%	453	63.4%	440	64.6%	410	60.7%	
**Place of residence**									0.302
A small town or village	383	51.3%	392	50.8%	395	54.8%	389	54.3%	
City	363	48.7%	380	49.2%	326	45.2%	328	45.7%	
**Marital status**									<0.001 **
Married	452	60.6%	482	62.4%	505 ^	70.0% ^	504	70.3%	
Single	294	39.4%	290	37.6%	216 *	30.0% *	213	29.7%	
**Number of family members**									0.403
Two or more	664	89.0%	668	86.5%	623	86.4%	624	87.0%	
One	82	11.0%	104	13.5%	98	13.6%	93	13.0%	
**With children under 18 years old**									0.102
Yes	297	39.8%	337	43.7%	286	39.7%	321	44.8%	
No	449	60.2%	435	56.3%	435	60.3%	396	55.2%	
**Employment**									0.496
Employed	534	76.6%	553	77.6%	497	74.3%	512	77.1%	
Unemployed	163	23.4%	160	22.4%	172	25.7%	152	22.9%	
**Income**									0.268
Higher	367	63.2%	385	63.8%	386	65.9%	358	60.4%	
Lower	214	36.8%	218	36.2%	200	34.1%	235	39.6%	
**Food selection criteria**									<0.001 **
Other	559	76.4%	644 ^	85.8% ^	574 *	80.5% *	552	78.1%	
To strengthen health	173	23.6%	107 *	14.2% *	139 ^	19.5% ^	155	21.9%	
**Severity of COVID-19**									0.296
Suffered from an asymptomatic or a mild form of COVID-19 (or did not have COVID-19 at all)	702	94.1%	726	94.0%	674	93.5%	659	91.9%	
Suffered from a severe form of COVID-19	44	5.9%	46	6.0%	47	6.5%	58	8.1%	
**Subjective assessment of health status**									0.075
Positive	660	89.2%	687	90.2%	614 *	86.1% *	641	89.4%	
Negative	80	10.8%	75	9.8%	99 ^	13.9% ^	76	10.6%	
**Subjective assessment of nutrition**									0.148
Positive	617	83.5%	607	80.0%	574	80.6%	559	79.0%	
Negative	122	16.5%	152	20.0%	138	19.4%	149	21.0%	
**Consumption of dietary supplements**									0.016 **
No	180	24.4%	235 ^	30.7% ^	222	30.9%	214	29.9%	
Yes	558	75.6%	530 *	69.3% *	496	69.1%	502	70.1%	
**Health-consciousness in product choice**									<0.001 **
Pays attention to buying healthy products	579	77.6%	496 *	64.2% *	542 ^	75.2% ^	515	71.8%	
Does not care about the healthiness of products	167	22.4%	276 ^	35.8% ^	179 *	24.8% *	202	28.2%	

^ a significantly higher prevalence (*p* < 0.05) compared to the sample collected a year ago (2021 vs. 2022, 2022 vs. 2023, 2023 vs. 2024); * a significantly lower prevalence (*p* < 0.05) compared to the sample collected a year ago (2021 vs. 2022, 2022 vs. 2023, 2023 vs. 2024); ** a significant difference (*p* < 0.05) among all four samples (2021, 2022, 2023, and 2024).

**Table 4:** Distribution of the respondents engaged in exercise and sports (regular or irregular) by social and demographic factors in four samples.

Factor	Sample of 2021		Sample of 2022		Sample of 2023		Sample of 2024		*p*-Value
	*n*	%	*n*	%	*n*	%	*n*	%	
**Sex**									0.729
Male	405	50.0%	360	48.4%	405	50.6%	409	51.1%	
Female	405	50.0%	384	51.6%	395	49.4%	391	48.9%	
**Age**									0.966
41 years old or younger	408	50.4%	378	50.8%	413	51.6%	409	51.1%	
42 years old or older	402	49.6%	366	49.2%	387	48.4%	391	48.9%	
**Education**									0.018 **
Non-university education	169	22.0%	189 ^	26.4% ^	189	24.7%	223	28.9%	
University education	598	78.0%	527 *	73.6% *	577	75.3%	549	71.1%	
**Place of residence**									0.938
A small town or village	379	46.8%	345	46.4%	370	46.3%	362	45.3%	
City	431	53.2%	399	53.6%	430	53.8%	438	54.8%	
**Marital status**									<0.001 **
Married	497	61.4%	426	57.3%	541 ^	67.6% ^	524	65.5%	
Single	313	38.6%	318	42.7%	259 *	32.4% *	276	34.5%	
**Number of family members**									0.082
Two or more	714	88.1%	624 *	83.9% *	677	84.6%	682	85.3%	
One	96	11.9%	120 ^	16.1% ^	123	15.4%	118	14.8%	
**With children under 18 years old**									0.949
Yes	305	37.7%	277	37.2%	308	38.5%	307	38.4%	
No	505	62.3%	467	62.8%	492	61.5%	493	61.6%	
**Employment**									0.009 **
Employed	628	82.8%	532*	76.9% *	608^	81.2% ^	610	83.1%	
Unemployed	130	17.2%	160^	23.1% ^	141*	18.8%*	124	16.9%	
**Income**									0.180
Higher	458	72.8%	390	69.3%	459	72.4%	433	68.1%	
Lower	171	27.2%	173	30.7%	175	27.6%	203	31.9%	
**Food selection criteria**									<0.001 **
Other	463	58.2%	533 ^	73.3% ^	536 *	68.0% *	517	65.6%	
Health strengthening	332	41.8%	194 *	26.7% *	252 ^	32.0% ^	271	34.4%	
**Severity of COVID-19**									0.257
Suffered from an asymptomatic or a mild form of COVID-19 (or did not have COVID-19 at all)	774	95.6%	698	93.8%	756	94.5%	747	93.4%	
Suffered from a severe form of COVID-19	36	4.4%	46	6.2%	44	5.5%	53	6.6%	
**Subjective assessment of health status**									0.301
Positive	766	95.0%	689	93.0%	755	94.7%	757	94.6%	
Negative	40	5.0%	52	7.0%	42	5.3%	43	5.4%	
**Subjective assessment of nutrition**									0.003 **
Positive	753	93.4%	673	90.9%	704	88.4%	709	89.2%	
Negative	53	6.6%	67	9.1%	92	11.6%	86	10.8%	
**Consumption of dietary supplements**									0.010 **
No	142	17.6%	171 ^	23.2% ^	148 *	18.7% *	180	22.6%	
Yes	666	82.4%	567 *	76.8% *	645^	81.3% ^	617	77.4%	
**Health-consciousness in product choice**									0.241
Pays attention to buying healthy products	705	87.0%	623	83.7%	674	84.3%	686	85.8%	
Does not care about the healthiness of products	105	13.0%	121	16.3%	126	15.8%	114	14.3%	

^ a significantly higher prevalence (*p* < 0.05) compared to the sample collected a year ago (2021 vs. 2022, 2022 vs. 2023, 2023 vs. 2024); * a significantly lower prevalence (*p* < 0.05) compared to the sample collected a year ago (2021 vs. 2022, 2022 vs. 2023, 2023 vs. 2024); ** a significant difference (*p* < 0.05) among all four samples (2021, 2022, 2023, and 2024).

**Table 5:** Distribution of the respondents engaged in regular exercise and sports by social and demographic factors in four samples.

Factor	Sample of 2021		Sample of 2022		Sample of 2023		Sample of 2024		*p*-Value
	*n*	%	*n*	%	*n*	%	*n*	%	
**Sex**									0.352
Male	95	54.3%	93	50.5%	99	51.8%	106	45.7%	
Female	80	45.7%	91	49.5%	92	48.2%	126	54.3%	
**Age**									0.609
41 years old or younger	107	61.1%	100	54.3%	112	58.6%	137	59.1%	
42 years old or older	68	38.9%	84	45.7%	79	41.4%	95	40.9%	
**Education**									0.044 **
Non-university education	30	18.0%	54 ^	30.3% ^	39 *	21.3% *	56	24.8%	
University education	137	82.0%	124 *	69.7% *	144 ^	78.7% ^	170	75.2%	
**Place of residence**									0.897
A small town or village	66	37.7%	72	39.1%	79	41.4%	94	40.5%	
City	109	62.3%	112	60.9%	112	58.6%	138	59.5%	
**Marital status**									0.044 **
Married	110	62.9%	103	56.0%	134 ^	70.2% ^	147	63.4%	
Single	65	37.1%	81	44.0%	57 *	29.8% *	85	36.6%	
**Number of family members**									0.832
Two or more	149	85.1%	159	86.4%	161	84.3%	193	83.2%	
One	26	14.9%	25	13.6%	30	15.7%	39	16.8%	
**With children under 18 years old**									0.864
Yes	72	41.1%	74	40.2%	73	38.2%	98	42.2%	
No	103	58.9%	110	59.8%	118	61.8%	134	57.8%	
**Employment**									0.019 **
Employed	135	82.8%	125 *	71.4% *	153 ^	83.2% ^	172	81.1%	
Unemployed	28	17.2%	50^	28.6% ^	31 *	16.8% *	40	18.9%	
**Income**									0.013 **
Higher	111	81.0%	89 *	65.0% *	112 ^	78.3% ^	128	74.0%	
Lower	26	19.0%	48 ^	35.0% ^	31 *	21.7% *	45	26.0%	
**Food selection criteria**									0.024 **
Other	90	51.7%	116 ^	65.5% ^	122	64.6%	146	63.8%	
Health strengthening	84	48.3%	61 *	34.5% *	67	35.4%	83	36.2%	
**Severity of COVID-19**									0.512
Suffered from an asymptomatic or a mild form of COVID-19 (or did not have COVID-19 at all)	168	96.0%	173	94.0%	185	96.9%	219	94.4%	
Suffered from a severe form of COVID-19	7	4.0%	11	6.0%	6	3.1%	13	5.6%	
**Subjective assessment of health status**									0.449
Positive	170	97.1%	175	95.6%	185	97.4%	228	98.3%	
Negative	5	2.9%	8	4.4%	5	2.6%	4	1.7%	
**Subjective assessment of nutrition**									0.177
Positive	167	95.4%	169	92.3%	172	90.1%	217	94.3%	
Negative	8	4.6%	14	7.7%	19	9.9%	13	5.7%	
**Consumption of dietary supplements**									0.165
No	33	18.9%	45	24.7%	31 *	16.5% *	54	23.4%	
Yes	142	81.1%	137	75.3%	157 ^	83.5% ^	177	76.6%	
**Health-consciousness in product choice**									0.426
Pays attention to buying healthy products	152	86.9%	154	83.7%	171	89.5%	200	86.2%	
Does not care about the healthiness of products	23	13.1%	30	16.3%	20	10.5%	32	13.8%	

^ a significantly higher prevalence (*p* < 0.05) compared to the sample collected a year ago (2021 vs. 2022, 2022 vs. 2023, 2023 vs. 2024); * a significantly lower prevalence (*p* < 0.05) compared to the sample collected a year ago (2021 vs. 2022, 2022 vs. 2023, 2023, vs. 2024); ** a significant difference (*p* < 0.05) among all four samples (2021, 2022, 2023, and 2024).

**Table 6:** Distribution of respondents by engagement in exercise and sports (regular or irregular) and sociodemographic factors in four samples. Note: This table depicts only figures and row percentages of respondents who distinctly, for each sample, reported engagement in exercise and sports (regular or irregular).

Factor	Sample of 2021		Sample of 2022		Sample of 2023		Sample of 2024	
	*n*	%	*n*	%	*n*	%	*n*	%
**Sex**								
Male	405	53.8%	360	49.3%	405 ^h^	55.6%	409 ^h^	55.8%
Female	405	50.4%	384	48.9%	395 *	49.9%	391 *	49.9%
**Age**								
41 years old or younger	408	53.7%	378	49.7%	413	54.7%	409	54.0%
42 years old or older	402	50.5%	366	48.5%	387	50.5%	391	51.5%
**Education**								
Non-university education	169 *	40.0%	189 *	42.0%	189 *	44.0%	223 *	45.6%
University education	598 ^h^	58.1%	527 ^h^	53.8%	577 ^h^	56.7%	549 ^h^	57.2%
**Place of residence**								
A small town or village	379	49.7%	345	46.8%	370 *	48.4%	362 *	48.2%
City	431	54.3%	399	51.2%	430 ^h^	56.9%	438 ^h^	57.2%
**Marital status**								
Married	497	52.4%	426 *	46.9%	541	51.7%	524 *	51.0%
Single	313	51.6%	318 ^h^	52.3%	259	54.5%	276 ^h^	56.4%
**Number of family members**								
Two or more	714	51.8%	624	48.3%	677	52.1%	682	52.2%
One	96	53.9%	120	53.6%	123	55.7%	118	55.9%
**With children under 18 years old**								
Yes	305	50.7%	277 *	45.1%	308	51.9%	307 *	48.9%
No	505	52.9%	467 ^h^	51.8%	492	53.1%	493 ^h^	55.5%
**Employment**								
Employed	628 ^h^	54.0%	532	49.0%	608 ^h^	55.0%	610 ^h^	54.4%
Unemployed	130 *	44.4%	160	50.0%	141 *	45.0%	124 *	44.9%
**Income**								
Higher	458 ^h^	55.5%	390 ^h^	50.3%	459 ^h^	54.3%	433 ^h^	54.7%
Lower	171 *	44.4%	173 *	44.2%	175 *	46.7%	203 *	46.3%
**Food selection criteria**								
Other	463 *	45.3%	533 *	45.3%	536 *	48.3%	517 *	48.4%
Health strengthening	332 ^h^	65.7%	194 ^h^	64.5%	252 ^h^	64.5%	271 ^h^	63.6%
**Severity of COVID-19**								
Suffered from an asymptomatic or a mild form of COVID-19 (or did not have COVID-19 at all)	774	52.4%	698	49.0%	756	52.9%	747	53.1%
Suffered from a severe form of COVID-19	36	45.0%	46	50.0%	44	48.4%	53	47.7%
**Subjective assessment of health status**								
Positive	766 ^h^	53.7%	689 ^h^	50.1%	755 ^h^	55.1%	757 ^h^	54.1%
Negative	40 *	33.3%	52 *	40.9%	42 *	29.8%	43 *	36.1%
**Subjective assessment of nutrition**								
Positive	753 ^h^	55.0%	673 ^h^	52.6%	704 ^h^	55.1%	709 ^h^	55.9%
Negative	53 *	30.3%	67 *	30.6%	92 *	40.0%	86 *	36.6%
**Consumption of dietary supplements**								
No	142 *	44.1%	171 *	42.1%	148 *	40.0%	180 *	45.7%
Yes	666 ^h^	54.4%	567 ^h^	51.7%	645 ^h^	56.5%	617 ^h^	55.1%
**Health-consciousness in product choice**								
Pays attention to buying healthy products	705 ^h^	54.9%	623 ^h^	55.7%	674 ^h^	55.4%	686 ^h^	57.1%
Does not care about the healthiness of products	105 *	38.6%	121 *	30.5%	126 *	41.3%	114 *	36.1%

^h^—a significantly higher proportion (*p* < 0.05) compared to those reporting no engagement in physical activity; * a significantly lower proportion (*p* < 0.05) compared to those reporting no engagement in physical activity.

**Table 7:** Binary logistic regression analysis to identify the independent factors associated with engagement in exercise and sports (regular or irregular).

Factor	Odds Ratio (95% CI)	*p*-Value
Being male	1.26 (1.115–1.425)	<0.001
Being single	1.223 (1.067–1.402)	0.004
Absence of children under 18 years of age	1.222 (1.061–1.408)	0.005
Selecting foods for health strengthening	1.736 (1.5–2.009)	<0.001
Positive assessment of nutrition	2.069 (1.726–2.48)	<0.001
Positive assessment of health status	1.746 (1.385–2.201)	<0.001
Use of dietary supplements	1.469 (1.268–1.701)	<0.001
Pays attention to buying healthy products	1.478 (1.26–1.733)	<0.001
University education	1.58 (1.369–1.824)	<0.001
Higher income	1.134 (0.984–1.308)	0.083
Increase in age (per year)	0.986 (0.982–0.991)	<0.001

Negelkerke R Square 0.110, Cox and Snell R Square 0.082, Hosmer and Lemeshow Test *p* = 0.980, overall, correctly predicted percentage 62.1 (with the cut value 0.5). Odds ratios were adjusted for age, sex, marital status, presence of children under 18 years of age, food selection criteria, self-assessed quality of nutrition, self-assessed health status, use of dietary supplements, health consciousness in product choice, education level, and income.

**Table 8:** Distribution of respondents by engagement in regular exercise and sports and sociodemographic factors in four samples. Note: This table shows only figures and row percentages of the respondents who distinctly, for each sample, reported engagement in regular exercise and sports.

Factor	Sample of 2021		Sample of 2022		Sample of 2023		Sample of 2024	
	*n*	%	*n*	%	*n*	%	*n*	%
**Sex**								
Male	95	12.6%	93	12.7%	99	13.6%	106	14.5%
Female	80	10.0%	91	11.6%	92	11.6%	126	16.1%
**Age**								
41 years old or younger	107 ^h^	14.1%	100	13.1%	112 ^h^	14.8%	137 ^h^	18.1%
42 years old or older	68 *	8.5%	84	11.1%	79 *	10.3%	95 *	12.5%
**Education**								
Non-university education	30 *	7.1%	54	12.0%	39 *	9.1%	56 *	11.5%
University education	137 ^h^	13.3%	124	12.7%	144 ^h^	14.2%	170 ^h^	17.7%
**Place of residence**								
A small town or village	66 *	8.7%	72 *	9.8%	79 *	10.3%	94 *	12.5%
City	109 ^h^	13.7%	112 ^h^	14.4%	112 ^h^	14.8%	138 ^h^	18.0%
**Marital status**								
Married	110	11.6%	103	11.3%	134	12.8%	147	14.3%
Single	65	10.7%	81	13.3%	57	12.0%	85	17.4%
**Number of family members**								
Two or more	149	10.8%	159	12.3%	161	12.4%	193	14.8%
One	26	14.6%	25	11.2%	30	13.6%	39	18.5%
**With children under 18 years old**								
Yes	72	12.0%	74	12.1%	73	12.3%	98	15.6%
No	103	10.8%	110	12.2%	118	12.7%	134	15.1%
**Employment**								
Employed	135	11.6%	125	11.5%	153	13.8%	172	15.3%
Unemployed	28	9.6%	50	15.6%	31	9.9%	40	14.5%
**Income**								
Higher	111 ^h^	13.5%	89	11.5%	112 ^h^	13.3%	128 ^h^	16.2%
Lower	26 *	6.8%	48	12.3%	31 *	8.3%	45 *	10.3%
**Food selection criteria**								
Other	90 *	8.8%	116 *	9.9%	122 *	11.0%	146 *	13.7%
Health strengthening	84 ^h^	16.6%	61 ^h^	20.3%	67 ^h^	17.1%	83 ^h^	19.5%
**Severity of COVID-19**								
Suffered from an asymptomatic or a mild form of COVID-19 (or did not have COVID-19 at all)	168	11.4%	173	12.1%	185	12.9%	219	15.6%
Suffered from a severe form of COVID-19	7	8.8%	11	12.0%	6	6.6%	13	11.7%
**Subjective assessment of health status**								
Positive	170 ^h^	11.9%	175 ^h^	12.7%	185 ^h^	13.5%	228 ^h^	16.3%
Negative	5 *	4.2%	8 *	6.3%	5 *	3.5%	4 *	3.4%
**Subjective assessment of nutrition**								
Positive	167 ^h^	12.2%	169 ^h^	13.2%	172 ^h^	13.5%	217 ^h^	17.1%
Negative	8 *	4.6%	14 *	6.4%	19 *	8.3%	13 *	5.5%
**Consumption of dietary supplements**								
No	33	10.2%	45	11.1%	31 *	8.4%	54	13.7%
Yes	142	11.6%	137	12.5%	157 ^h^	13.8%	177	15.8%
**Health-consciousness in product choice**								
Pays attention to buying healthy products	152	11.8%	154 ^h^	13.8%	171 ^h^	14.1%	200 ^h^	16.7%
Does not care about the healthiness of products	23	8.5%	30 *	7.6%	20 *	6.6%	32 *	10.1%

^h^—a significantly higher proportion (*p* < 0.05) compared to those reporting no engagement in regular physical activity; * a significantly lower proportion (*p* < 0.05) compared to those reporting no engagement in regular physical activity.

**Table 9:** Binary logistic regression analysis to identify the independent factors associated with engagement in regular exercise and sports.

Factor	Odds Ratio (95% CI)	*p*-Value
Selecting foods for health strengthening	1.806 (1.483–2.2)	<0.001
Positive assessment of nutrition	2.324 (1.633–3.308)	<0.001
Positive assessment of health status	2.964 (1.71–5.138)	<0.001
Use of dietary supplements	1.229 (0.974–1.551)	0.083
Pays attention to buying healthy products	1.25 (0.954–1.637)	0.105
University education	1.441 (1.15–1.806)	0.001
Higher income	1.217 (0.981–1.511)	0.075
Later survey year	1.106 (1.021–1.197)	0.014
Urban residence	1.485 (1.229–1.793)	<0.001
Increase in age (per year)	0.977 (0.97–0.984)	<0.001

Negelkerke R Square 0.083, Cox and Snell R Square 0.044, Hosmer and Lemeshow Test *p* = 0.829, overall correctly predicted percentage 87.5 (with the cut value 0.5). Odds ratios were adjusted for age, place of residence, food selection criteria, self-assessed quality of nutrition, self-assessed health status, use of dietary supplements, health consciousness in product choice, education level, income, and survey year.

## Data Availability

The raw data supporting the conclusions of this article will be made available by the authors on request.
